# Field Evidence for Asymmetric Regulation of Wheat Streak Mosaic Virus and Triticum Mosaic Virus Across the Wheat–Wheat Curl Mite Interface

**DOI:** 10.3390/insects17050459

**Published:** 2026-04-28

**Authors:** Saurabh Gautam, Kiran R. Gadhave

**Affiliations:** 1 Alliance of Pest Control Districts, Tulare, CA 93274, USA; saurabh@apcd.ca.gov; 2 Texas A&M AgriLife Research, High Plains Research and Extension Center, Canyon, TX 79015, USA; 3 Department of Entomology, Texas A&M University, College Station, TX 77843, USA

**Keywords:** wheat streak mosaic, wheat curl mite, *Wsm1* gene, *Wsm2* gene, mixed virus infection, plant–vector interactions

## Abstract

Wheat streak mosaic (WSM), historically attributed to wheat streak mosaic virus (WSMV) and transmitted by the wheat curl mite (WCM; *Aceria tosichella*), remains a major cause of yield loss in the Texas High Plains. In recent years, Triticum mosaic virus (TriMV), also transmitted by WCM, has emerged as an increasingly important component of the WSM disease complex. Under field conditions, TriMV is most frequently detected in mixed infections with WSMV. Management of WSM relies primarily on resistant cultivars carrying genes such as *Wsm1* or *Wsm2*. Although synergistic interactions between WSMV and TriMV have been documented under controlled conditions, their dynamics during natural field infections—particularly during the latent phase between initial infection and symptom development—remain poorly understood. Moreover, the extent to which host genotype influences virus–virus interactions and vector acquisition dynamics in the field has not been fully resolved. Replicated field trials conducted over two growing seasons were used to quantify temporal accumulation patterns and relative ratios of WSMV and TriMV in susceptible (TAM 304) and resistant cultivars differing in resistance source (BT [*Wsm1*] and Joe [*Wsm2*]) under natural disease spread. WSMV remained the predominant virus in mixed infections across cultivars, sampling times, and disease stages. However, as plants aged and entered senescence, WSMV titers declined more rapidly than TriMV titers, resulting in a progressive reduction in the WSMV-to-TriMV ratio. From early infection through disease development, the *Wsm1* cultivar (BT) consistently supported significantly lower TriMV accumulation than the *Wsm2* cultivar (Joe), providing a mechanistic explanation for the comparatively stronger disease suppression associated with WSM. Mites feeding on BT also acquired lower TriMV titers. Although viral concentrations in wheat tissue were strongly correlated with those detected in feeding mites, substantial differences in plant-level WSMV-to-TriMV ratios among cultivars were not mirrored within the vector. These findings indicate that while host resistance regulates absolute virus accumulation, vector-associated factors may influence the relative proportions of viruses detected following acquisition, with important implications for WSM epidemiology and resistance deployment in field systems.

## Simple Summary

Wheat streak mosaic (WSM) is a damaging viral disease of wheat transmitted by the wheat curl mite. It is caused primarily by wheat streak mosaic virus (WSMV), but Triticum mosaic virus (TriMV) often occurs alongside it and can worsen disease severity. In this two-year field study, we tracked how these two viruses accumulated over time in susceptible and resistant wheat cultivars under natural conditions. WSMV was generally more abundant, but its levels declined rapidly as plants aged, while TriMV declined more gradually. Wheat cultivars carrying the *Wsm1* resistance gene limited TriMV more effectively than that carrying *Wsm2*, resulting in lower virus levels in both plants and feeding mites. Interestingly, although virus ratios differed among wheat cultivars, mites feeding on them carried similar proportions of the two viruses. This suggests that while plant resistance reduces overall virus levels, processes within the mite may regulate how much of each virus is transmitted. Understanding these interactions helps improve breeding and management strategies for wheat streak mosaic.

## 1. Introduction

In the Texas Panhandle, hard red winter wheat—grown on approximately three million acres for both grain production and winter cattle grazing—faces persistent pressure from wheat streak mosaic (WSM) [1]. This disease complex is caused by wheat streak mosaic virus (WSMV), Triticum mosaic virus (TriMV), or their co-infection, with both viruses transmitted by the wheat curl mite (*Aceria tosichella*). Although WSMV has historically been the dominant pathogen, TriMV has expanded rapidly in the region since 2008 and is now frequently detected in mixed infections in commercial fields [2,3]. WSM severely reduces plant vigor, causing mosaic symptoms, chlorotic streaking, and stunting; early-season infection can result in plant death and substantial forage and grain losses. Reported economic losses in 2010 ranged from $118.1 to $464.5 per hectare [1]. Irrigated systems, which account for roughly one-third of regional wheat acreage, are particularly vulnerable due to compromised water use efficiency in infected plants [4].

Production practices further influence disease risk. Wheat is planted from late August through mid-September to maximize fall forage, but early planting increases exposure to the “green bridge,” a continuum of volunteer wheat and grassy hosts that sustains wheat curl mite (WCM) populations and virus reservoirs [5,6,7,8,9,10]. Wind-dispersed mites move from surrounding vegetation into newly established fields, often initiating infections at field margins [11]. As temperatures rise in spring, WCM generation time shortens to 7–10 days, accelerating population growth and virus spread [12,13]. Management options are limited: no acaricides are registered for effective in-season control, and host resistance—primarily through *Wsm1* or *Wsm2*—can be temperature sensitive, often diminishing above 27 °C [14,15,16].

Mixed infection by WSMV and TriMV is associated with synergistic interactions that increase viral titers and exacerbate symptom severity relative to single infections [17,18]. While viral accumulation has been well characterized in late-stage symptomatic plants, less is known about early infection dynamics under field conditions, particularly regarding how host genotype influences virus–virus interactions and vector acquisition during disease establishment. Because winter wheat is a long-season crop in which virus accumulation and disease severity are strongly influenced by host developmental stage, particularly the timing of infection relative to vegetative growth, reproductive development, and senescence [8,9], understanding these early spatiotemporal dynamics is critical for epidemiological modeling and resistance deployment.

In the case of WSM, infection occurring during early vegetative growth typically results in greater virus accumulation, more severe symptom expression, and larger yield losses than infections established near reproductive stages or during senescence. Consequently, temporal patterns of virus accumulation must be interpreted in the context of wheat phenology, as viral titers often increase during active vegetative growth and decline as plants transition to grain fill and senescence. Understanding how WSMV and TriMV accumulate across developmental stages is therefore essential for interpreting field-based disease dynamics and for evaluating the efficacy of resistance genes under natural epidemic conditions.

The present study evaluated seasonal changes in WSMV and TriMV accumulation and relative ratios in susceptible and resistant wheat cultivars as disease spread naturally from a trap wheat strip. We quantified viral titers in both plant tissues and feeding WCM populations to assess how host genotype and infection timing influence virus–virus and virus–vector interactions under field conditions.

## 2. Materials and Methods

The Field experiments were conducted over two consecutive winter wheat growing seasons (2021–2022 and 2022–2023) at Bushland, Texas, under a center-pivot irrigation system. During the 2021–2022 season, a 9 m-wide trap strip of TAM 304 wheat cultivar (hard red winter wheat cultivar susceptible to WSM and WCM) was established along the southern edge of the experimental field in July 2021 to serve as a source of WCM and mite-transmitted viruses. Experimental plots were drill-seeded in early September 2021 and arranged perpendicular to the trap strip, extending northward to capture spatial gradients of pathogen dispersal. In accordance with regional production practices, plots consisted of 20 rows spaced 19 cm apart and extended approximately 300 m (980 ft) in length. This experimental design enabled assessment of the longitudinal spread of WCM and viruses from a localized southern inoculum source under commercial-scale production conditions [11].

Three wheat cultivars were evaluated: the susceptible cultivar TAM 304, and two single-gene resistant cultivars, Breakthrough (BT; containing *Wsm1* gene) and Joe (containing *Wsm2* gene). Cultivars were planted in three replications arranged in a randomized complete block design. Prior to symptom development, flag markers were installed in February 2022 at 100-ft intervals along each replication (locations L1–L9), with L1 positioned closest to the trap strip. Disease symptoms first appeared in the trap strip between late February and early April 2022. Plant and wheat curl mite samples collected at that time confirmed the presence of mixed infections with WSMV and TriMV. High Plains wheat mosaic virus (HPWMoV, another WCM transmitted virus) was not detected and was therefore excluded from subsequent analyses of experimental plot samples. As disease spread progressed—typically from the field margins inward due to wind-mediated dispersal of WCM—plant sampling was conducted weekly at each flagged location for seven consecutive weeks (second week of April to the last week of May 2022). At each sampling event, a single tiller was destructively collected from the plant nearest to the flag within the row, and a different plant was sampled each week to avoid repeated sampling of individual plants. Sampling during the spatiotemporal analysis was not limited to visibly symptomatic plants. When disease symptoms were not yet apparent at a given location, plants exhibiting evidence of WCM infestation were preferentially selected when present. At locations and early sampling time points where neither visible disease symptoms nor mite infestation were observed, a tiller was collected from the plant closest to the flagged location within the row. This approach was most commonly applied during the initial sampling weeks, when disease and mite populations had not yet advanced into interior portions of the field. The sampling strategy was designed to characterize virus accumulation dynamics during epidemic establishment rather than to estimate field-level disease incidence or prevalence.

The experimental unit for plant-level analyses was the flagged location within each replication. One plant sample per cultivar was collected at each flagged location during each weekly sampling event. Thus, for each sampling week, nine plant samples per cultivar were collected per replication, resulting in 27 samples per cultivar per week across three replications. Over the seven-week sampling period, this design yielded a total of 189 plant samples per cultivar (27 samples × 7 weeks), for a cumulative total of 567 plant samples across all cultivars for the 2021–2022 season. Sampling during this period spanned key wheat developmental stages from late tillering through early grain fill. In 2022 growing season, plants started to enter senescence in mid-May 2022, coinciding with a decline in virus titers. Experimental plots were harvested at physiological maturity in second week of June 2022 in accordance with regional agronomic practices; however, yield data were not collected for this trial.

The experiment was repeated during the 2022–2023 growing season, with the trap strip again established in July 2022 and experimental plots planted in September 2022. Although wheat streak mosaic symptoms emerged in trap strips between late February and early April 2023, prolonged rainfall in April and May 2023 restricted field access, limiting sampling during early infection stages. Consequently, data collection during the second season was restricted to a three-week period from last week of May to mid-June 2023, when plants had reached late grain fill to early senescence. Unusually high soil moisture during 2023 also extended the growing season by approximately two to three weeks relative to typical regional condition. At each weekly sampling event, one plant per cultivar was sampled at each flagged location within each replication, yielding nine samples per cultivar per week (3 locations × 3 replications). Over the three-week sampling period, this resulted in a total of 27 plant samples per cultivar and 81 plant samples across all cultivars for the 2022–2023 season. Despite this temporal constraint, sampling captured the late-season decline phase of virus accumulation and provided independent validation of patterns observed in the first season. Experimental plots were harvested at physiological maturity in first week of July 2023, consistent with regional agronomic practices.

During the 2021–2022 growing season, virus accumulation and WSMV-to-TriMV titer ratios were also assessed in mites and the wheat plants they infested. Symptomatic plants exhibiting characteristic mite feeding injury were collected from the five flagged locations closest to the southern trap strip (L1–L5). Using a fine brush, 15–25 mites representing mixed developmental stages were collected from individual plants, and the specific plant tissues on which mites were actively feeding were sampled separately. Mites were pooled to obtain sufficient RNA for reliable downstream quantification, and all samples were stored at −80 °C until analysis. Mite samples were collected from five plants per cultivar across two sampling events (4 May 2022 and 9 May 2022), yielding *n* = 10 pooled mite samples per cultivar. Sampling was restricted to this period to coincide with peak wheat curl mite activity and the presence of uniformly symptomatic plants across multiple flagged locations, thereby providing a representative snapshot of virus acquisition during active epidemic development. At earlier or later time points, plants were largely asymptomatic and/or mite densities were insufficient to generate a spatially representative dataset reflecting field-level patterns of virus accumulation in mites relative to host plants.

Viral loads and WSMV-to-TriMV ratios in mite and plant samples were quantified by qRT-PCR as described previously by Tatineni et al. (2010) [19]. Total RNA was extracted from wheat tissue (200 mg) and pooled wheat curl mite samples using TRIzol™ reagent (Invitrogen, Thermo Fisher Scientific, Waltham, MA, USA) according to the manufacturer’s instructions. Samples were homogenized in TRIzol reagent, followed by phase separation with chloroform, isopropanol precipitation, and ethanol washing. RNA pellets were resuspended in nuclease-free water, and RNA concentration and purity were assessed using a NanoDrop spectrophotometer (Thermo Fisher Scientific). First-strand complementary DNA (cDNA) synthesis was performed using the Omniscript Reverse Transcription Kit (Qiagen, Hilden, Germany) following the manufacturer’s protocol. Reactions were carried out in a 20 µL volume using 500 ng to 1 µg of total RNA with random primers and incubated at 42 °C for 60 min, followed by enzyme inactivation at 95 °C for 5 min.

Quantitative real-time PCR (qRT-PCR) was performed using a TaqMan probe-based assay with Applied Biosystems™ TaqMan™ Fast Advanced Master Mix (Thermo Fisher Scientific) on an Applied Biosystems real-time PCR system. Virus-specific primers and probes targeting the coat protein (CP) genes of WSMV and TriMV, originally described by Tatineni et al. (2010) [19], were used for absolute quantification (Supplementary Table S1). Each reaction was conducted in a 25 µL volume containing 1× TaqMan Fast Advanced Master Mix, 500 nM of each primer, 250 nM probe, and 2 µL of diluted cDNA template. The thermal cycling conditions consisted of 50 °C for 2 min, 95 °C for 15 min, followed by 40 cycles of 95 °C for 15 s and 58 °C for 60 s, with fluorescence data collected during the annealing/extension step. All reactions were performed in technical duplicate or triplicate and included no-template controls to monitor contamination.

Absolute quantification of viral RNA copy number was performed using standard curves generated from ten-fold serial dilutions of plasmids containing the corresponding viral CP gene fragments. qPCR run quality parameters, including primer and probe sequences, amplification efficiency, coefficient of determination (R^2^), limits of detection (LOD), and limits of quantification (LOQ), are provided in Supplementary Table S1 in accordance with MIQE guidelines.

### Data Analysis

Data analyses were conducted in R version 3.6.0 [20]. Because of the significant rainfall event during the 2022–2023 season, data from the two growing seasons were analyzed separately. Spatiotemporal accumulation of WSMV and TriMV in plants, as well as their ratios, was analyzed using mixed-effects models [21]. Block was included as a random effect to account for field heterogeneity, and flagged location nested within replication was included as a random effect to account for spatial structure. Cultivars, time, and their interaction were treated as fixed effects. Virus accumulation and ratios in mites and corresponding plant tissues were analyzed using mixed-effects models, with replication included as a random effect and cultivar treated as a fixed effect. Statistical significance was determined at *p* < 0.05.

## 3. Results

### 3.1. Seasonal Virus Accumulation—Year 1 (2021–2022)

**Figure 1:**
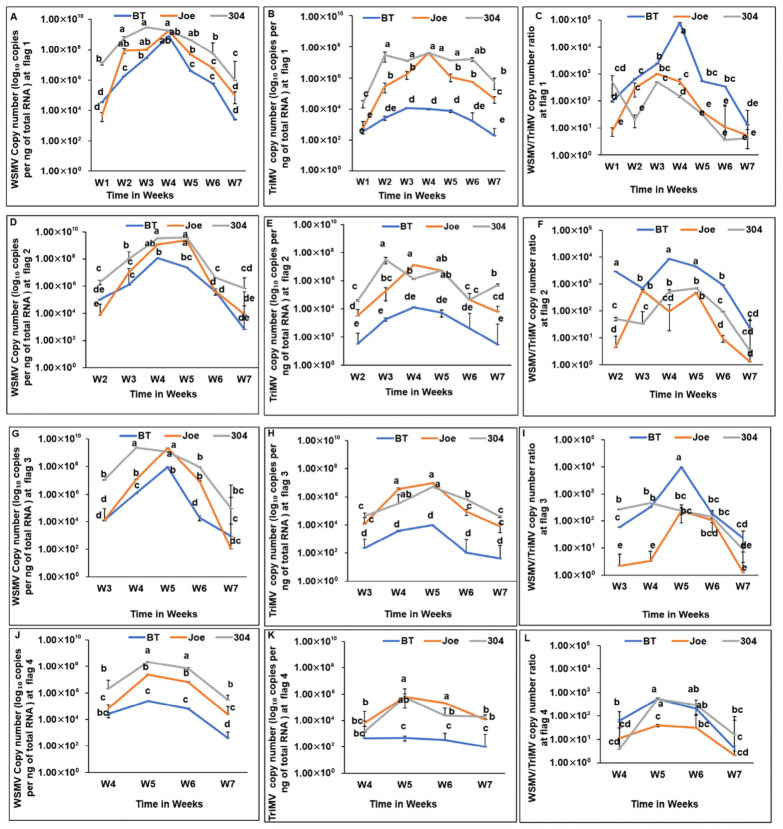
Spatiotemporal accumulation and titer ratios of wheat streak mosaic virus (WSMV) and Triticum mosaic virus (TriMV) in wheat cultivars during the 2021–2022 growing season. Panels are organized by flagged location beginning at the field edge adjacent to the trap strip. (**A**–**C**) Flagged location 1: (**A**) WSMV accumulation, (**B**) TriMV accumulation, and (**C**) WSMV-to-TriMV titer ratio. (**D**–**F**) Flagged location 2: (**D**) WSMV accumulation, (**E**) TriMV accumulation, and (**F**) WSMV-to-TriMV titer ratio. (**G**–**I**) Flagged location 3: (**G**) WSMV accumulation, (**H**) TriMV accumulation, and (**I**) WSMV-to-TriMV titer ratio. (**J**–**L**) Flagged location 4: (**J**) WSMV accumulation, (**K**) TriMV accumulation, and (**L**) WSMV-to-TriMV titer ratio. Data points represent mean viral RNA copy numbers (±SE) for cultivars Breakthrough (BT; *Wsm1*), Joe (*Wsm2*), and TAM 304, based on *n* = 3 biological replicates per cultivar per flagged location per sampling week (one plant per replication). Viral loads were quantified by qRT-PCR using absolute quantification of coat protein (CP) gene copies normalized per ng of total RNA and are shown on a logarithmic scale. Sampling was conducted weekly for seven consecutive weeks following local disease establishment. Consequently, the number of weeks shown differs among flagged locations because disease spread progressed spatially from the southern trap strip inward; locations farther from the trap strip became infected later. All nine flagged locations (L1–L9) were sampled; however, only L1–L4 are shown here because infection at L5–L9 occurred too late in the season to provide sufficient temporal resolution for visualization. Within each panel and sampling week, distinct letters indicate statistically significant differences in virus accumulation or ratios among cultivars (*p* < 0.05) at individual time points (e.g., higher WSMV accumulation in TAM304 relative to BT and Joe at week 1) and across time points (e.g., reduced WSMV accumulation in TAM304 at week 7 relative to week 1), based on mixed-effects models followed by post hoc pairwise comparisons.

Virus accumulation varied significantly over time in all cultivars during the first growing season (Figure 1). Across cultivar groups and sampling locations, WSMV and TriMV titers increased following initial infection, peaked at approximately 4–5 weeks, and subsequently declined as plants entered senescence. Consistent with this trend, the WSMV-to-TriMV ratio increased 2–3 weeks after infection at each location before declining later in the season, reflecting a comparatively sharper reduction in WSMV titers.

At later infection stages, WSMV accumulation was occasionally lower in BT (*Wsm1*) than in Joe (*Wsm2*), but this difference was significant only at specific flagged locations and was not consistently observed across the field. (Figure 1A–D). More notably, TriMV accumulation was significantly lower in BT than in Joe and TAM 304 across nearly all locations and sampling intervals (Figure 1E–H), with the exception of Week 1 at flag location 1 and Week 4 at flag location 4.

**Figure 2:**
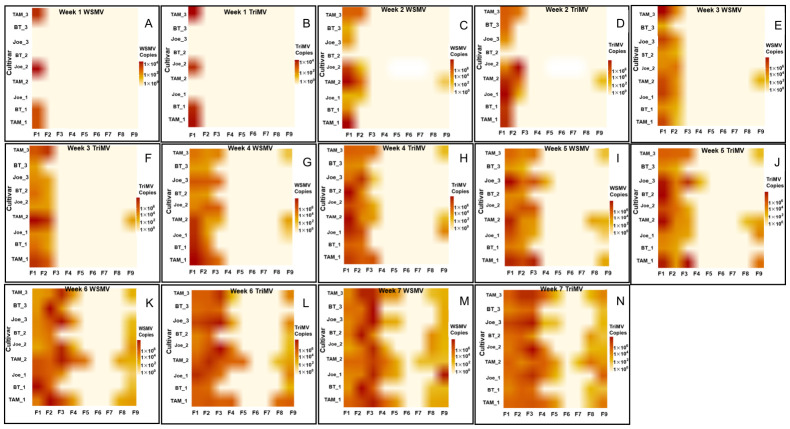
Heat maps (**A**–**N**) illustrating the spatiotemporal accumulation and titer ratios of WSMV and TriMV in wheat cultivars during the 2021–2022 growing season. Panels (**A**,**C**,**E**,**G**,**I**,**K**,**M**) represent WSMV accumulation, while panels (**B**,**D**,**F**,**H**,**J**,**L**,**N**) represent TriMV accumulation, corresponding to weeks 1 through 7, respectively. Heat maps are arranged by flagged location (F1–F9), beginning closest to the southern trap strip and extending northward into the field. Color intensity represents inverse hyperbolic sine (asinh)–transformed viral RNA copy number per ng of total RNA, derived from qRT PCR absolute quantification using plasmid based standard curves. The asinh transformation was applied solely for visualization purposes to improve contrast across a wide dynamic range of viral titers while retaining information from low abundance and near zero values. These heat maps provide a qualitative visualization of spatial and temporal trends in virus accumulation and are not intended to indicate statistical significance.

Spatially, infected plants were initially concentrated along the southern edge of the plots adjacent to the trap strip. Over time, infections progressed northward across the field (Figure 2). All sampled infected plants were positive for both WSMV and TriMV, indicating consistent mixed infections. Across sampling points, BT plants exhibited reduced accumulation of both viruses relative to Joe and TAM 304.

### 3.2. Seasonal Virus Accumulation—Year 2 (2022–2023)

**Figure 3:**
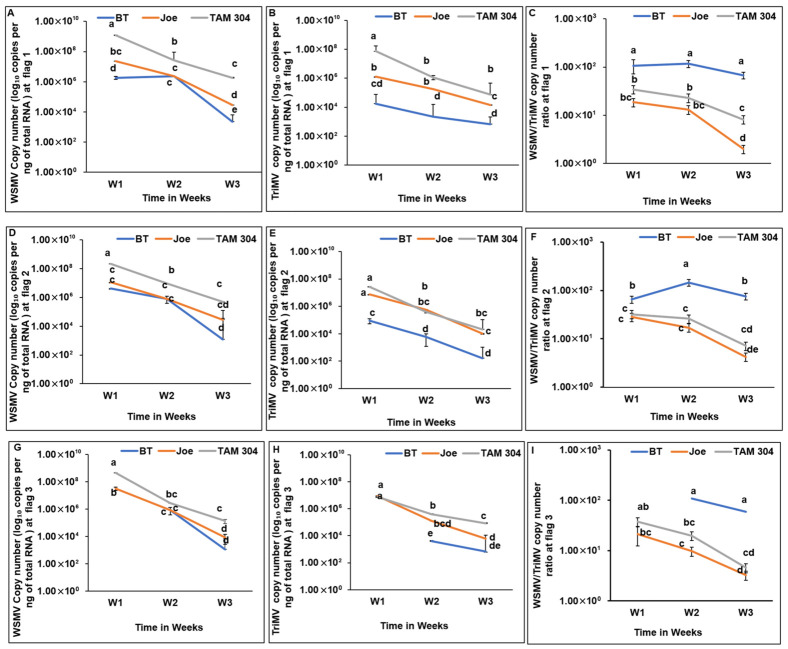
Spatiotemporal accumulation and titer ratios of wheat streak mosaic virus (WSMV) and Triticum mosaic virus (TriMV) in wheat cultivars during the 2022–2023 growing season. Panels are organized by flagged location beginning at the field edge adjacent to the southern trap strip. (**A**–**C**) Flagged location 1: (**A**) WSMV accumulation, (**B**) TriMV accumulation, and (**C**) WSMV-to-TriMV titer ratio. (**D**–**F**) Flagged location 2: (**D**) WSMV accumulation, (**E**) TriMV accumulation, and (**F**) WSMV-to-TriMV titer ratio. (**G**–**I**) Flagged location 3: (**G**) WSMV accumulation, (**H**) TriMV accumulation, and (**I**) WSMV-to-TriMV titer ratio. Data points represent mean viral RNA copy numbers (±SE) for cultivars Breakthrough (BT; *Wsm1*), Joe (*Wsm2*), and TAM 304, based on *n* = 3 biological replicates per cultivar per flagged location per sampling week (one plant per replication). Viral loads were quantified by qRT-PCR using absolute quantification of coat protein (CP) gene copies normalized per ng of total RNA and are presented on a logarithmic scale. Sampling was conducted at approximately one-week intervals over a three-week period (late May to mid-June 2023) following local disease establishment, corresponding to late grain fill through senescence. Although all nine flagged locations (L1–L9) were sampled, only L1–L3 are shown because disease establishment at more distant locations (L4–L9) occurred too late in the season to provide sufficient temporal resolution for visualization. Within each panel and sampling week, distinct letters indicate statistically significant differences in virus accumulation or ratios among cultivars (*p* < 0.05) at individual time points and across time points based on mixed-effects models followed by post hoc pairwise comparisons.

In the second season, data collection was limited to a three-week period (late May to mid-June). During this interval, WSMV and TriMV titers declined across all cultivars as plants senesced (Figure 3A–F). Consistent with Year 1, BT exhibited significantly lower TriMV accumulation across all locations and sampling times (Figure 3D–F). This reduction resulted in significantly higher WSMV-to-TriMV ratios in BT compared to Joe and TAM 304 (Figure 3G–I).

**Figure 4:**
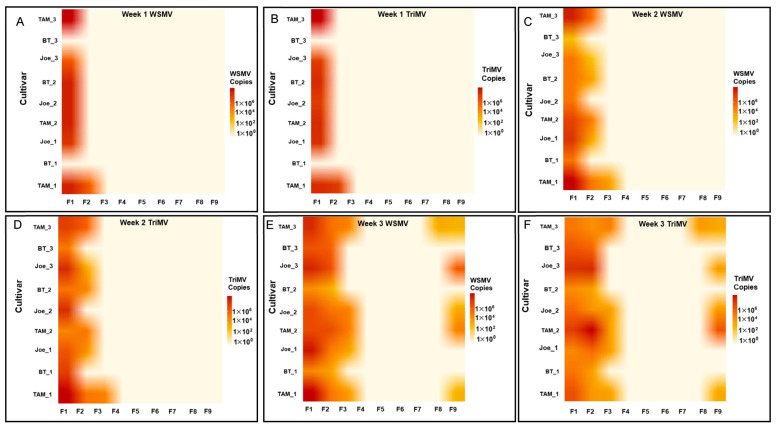
Heat maps (**A**–**F**) illustrating the spatiotemporal accumulation of wheat streak mosaic virus (WSMV) and Triticum mosaic virus (TriMV) across flagged locations and sampling weeks during the 2022–2023 growing season. Panels (**A**,**C**,**E**) represent WSMV accumulation, while panels (**B**,**D**,**F**) represent TriMV accumulation for weeks 1, 2, and 3, respectively. Heat maps are arranged by flagged location (F1–F3), beginning closest to the southern trap strip and extending northward into the field. Color intensity reflects inverse hyperbolic sine (asinh)–transformed viral RNA copy number per ng of total RNA, derived from qRT-PCR absolute quantification using plasmid-based standard curves. The asinh transformation was applied solely to improve visual contrast across a wide dynamic range of virus titers while retaining information from low-abundance and near-zero values. These heat maps provide a qualitative visualization of spatial and temporal trends in virus accumulation and are not intended to indicate statistical significance.

Spatial infection patterns mirrored those observed in the first season. Symptomatic plants were initially concentrated near the southern inoculum source and subsequently detected across the field (Figure 4A–F). All infected plants sampled during this season also showed mixed infection.

Full statistical outputs from the linear mixed-effects models, including F-statistics, degrees of freedom, and *p*-values for cultivar, time, and their interaction for both growing seasons, are provided in Supplementary Tables S2 and S3.

### 3.3. Virus Accumulation in Wheat Curl Mites

**Figure 5:**
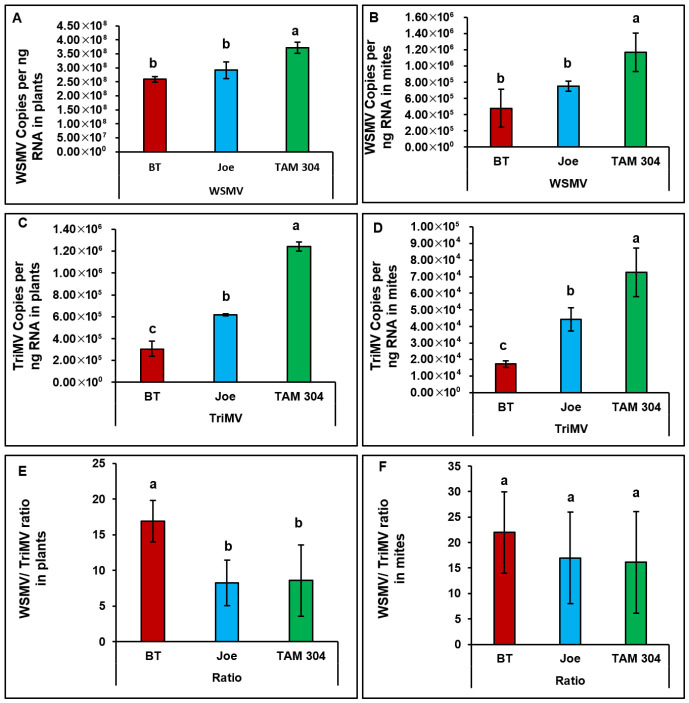
Bar graphs showing the accumulation and titer ratios of wheat streak mosaic virus (WSMV) and Triticum mosaic virus (TriMV) in both wheat cultivars (i.e., source) and the wheat curl mites (WCM; *Aceria tosichella*) that fed on each respective cultivar (i.e., sink). Symptomatic wheat plants displaying characteristic WCM feeding injury were collected from the five flagged locations most proximal to the trap strip. In the laboratory, 15–25 mites of mixed developmental stages and their associated feeding tissues were isolated using a fine brush and transferred into separate 1.5 mL microcentrifuge tubes. Virus quantification was performed via qRT-PCR using absolute quantification of coat protein (CP) gene copies against plasmid standards. The accompanying figures present the mean values and standard errors for WSMV titers in plants (**A**) and mites (**B**), TriMV titers in plants (**C**) and mites (**D**), and the corresponding WSMV-to-TriMV ratio in plants (**E**) and mites (**F**). Results are displayed on a logarithmic scale, with distinct letters indicating statistically significant differences (*p* < 0.05).

Virus accumulation differed among cultivars at the plant level (Figure 5A,C). For WSMV, titers were significantly higher in the susceptible cultivar TAM 304 than in the resistant cultivars, whereas no significant difference was detected between BT (*Wsm1*) and Joe (*Wsm2*). In contrast, TriMV accumulation differed significantly among all three cultivars, with lowest titers observed in BT compared with Joe and TAM 304 (Figure 5E).

Virus accumulation in mites feeding on infected plants followed the same relative pattern observed in plant tissues, indicating a strong association between source plant titers and mite viral load (Figure 5B,D). However, despite significant differences in WSMV-to-TriMV ratios among cultivars at the plant level, viral ratios within mites did not differ significantly among cultivars (Figure 5F).

## 4. Discussion

Wheat curl mite-transmitted wheat streak mosaic continues to cause substantial economic losses across the U.S. Great Plains. Although WSMV has historically been regarded as the principal causal agent of this disease complex, accumulating evidence indicates that TriMV is a major contributor to symptom development and is frequently detected in mixed infections [22,23]. Previous work demonstrated that WSMV and TriMV interact synergistically to intensify disease severity relative to single infections, independent of cultivar genotype [18]. This synergism, however, appears to be largely driven by TriMV, whose accumulation increases significantly in co-infected plants compared to singly infected plants [18].

In the present field study, the cultivar carrying the *Wsm1* gene (Breakthrough, BT) provided stronger protection against WSM than cultivars carrying *Wsm2* (Joe) or lacking resistance (TAM 304). This enhanced protection was consistently associated with reduced TriMV accumulation beginning early in infection and persisting through later disease stages, resulting in lower overall symptom severity (field observations). The consistently greater suppression of TriMV in *Wsm1* containing cultivars suggests fundamental differences in how these resistance genes modulate virus replication, movement, or host defense responses. Although the present study was not designed to directly resolve molecular mechanisms, prior work provides important context for interpreting these patterns. The *Wsm1* gene, introgressed from *Thinopyrum intermedium*, has been associated with broader and more durable resistance to wheat curl mite-transmitted viruses and is generally less prone to temperature dependent breakdown than *Wsm2* [15,24]. In contrast, resistance conferred by *Wsm2* is more temperature sensitive and has been linked to incomplete suppression of viral accumulation under both controlled and field conditions [14].

Although the molecular basis of *Wsm1*-mediated resistance remains unresolved, resistance introgressed from *T. intermedium* is thought to involve enhanced restriction of viral replication or systemic movement rather than complete immunity. In cereals, resistance to potyviruses is often associated with modulation of antiviral RNA silencing pathways or interference with long-distance virus movement, mechanisms that could differentially affect TriMV, a potyvirus, relative to WSMV, a tritimovirus, due to differences in genome organization, replication strategy, and movement protein function [25,26,27]. Because TriMV has been shown to play a central role in synergistic disease development during mixed infections [18,19], early and sustained suppression of TriMV replication or movement by *Wsm1* could disproportionately mitigate disease severity even when WSMV remains detectable. Determining whether *Wsm1* primarily restricts TriMV replication, limits long-distance movement, enhances host antiviral signaling, or disrupts virus–virus synergism will require targeted studies examining virus accumulation kinetics, systemic movement, and host defense pathway activation in genetically defined backgrounds.

Viral accumulation in wheat is shaped by host developmental stage, genetic background, vector movement, and environmental conditions [28]. During the 2021 and 2022 growing seasons in Bushland, Texas, temperatures increased steadily from April to June (daily highs 15–32 °C; daily lows 1–15 °C), remaining within ranges permissive for WSM development. Previously under controlled conditions (20–26 °C), cultivars carrying *Wsm1* exhibit moderate viral accumulation and mild-to-moderate synergistic disease, whereas susceptible cultivars support higher titers and severe symptom expression [19]. Consistent with those findings, TriMV replication in BT was slower than in Joe (*Wsm2*) and the susceptible cultivar under field conditions. Although WSMV accumulation was also reduced in BT at certain sampling points, consistent suppression was more apparent during later stages of infection. Collectively, these data suggest that reduced disease severity in *Wsm1*-containing cultivars primarily reflects early and sustained suppression of TriMV.

In mixed infections, viral titers are rarely additive and instead reflect synergistic or antagonistic interactions. Ratios exceeding unity are generally interpreted as evidence of synergism [29]. Because WSMV and TriMV belong to distinct taxonomic groups, their interactions within wheat are temporally dynamic and context dependent [30]. Previous studies showed that prior TriMV infection enhances WSMV accumulation, whereas established WSMV infection can delay TriMV replication [31]. In the current study, WSMV titers consistently exceeded TriMV titers across cultivars and sampling times. However, the WSMV-to-TriMV ratio declined over time due to a marked reduction in WSMV levels, indicating that host physiological age strongly modulates these interactions. Field infections likely arise from simultaneous or sequential mite-mediated inoculation, and possibly seed transmission [32]. Although precise infection timing could not be determined, the consistent detection of both viruses in mite populations from trap crops and field plots supports the likelihood of simultaneous inoculation. The presence of additional endemic wheat viruses in the region may further influence disease dynamics, warranting future studies addressing multi-virus interactions in field settings.

Earlier controlled studies demonstrated that viral accumulation in WCM is influenced by host genotype and infection status, with density-dependent relationships between virus titers in plants and mites [18]. The present field data corroborate this pattern, as mites feeding on plants with higher WSMV or TriMV titers accumulated correspondingly higher viral loads. However, in contrast to prior reports indicating higher WSMV-to-TriMV ratios in mites than in host tissue [3], no significant differences in viral ratios between mites and their source plants were detected in this study.

This discrepancy likely reflects methodological and scale-dependent differences between studies. Earlier work relied primarily on single-mite analyses conducted under controlled conditions, which revealed substantial inter-individual variability in virus acquisition. In contrast, mite samples in the present study were pooled and collected under natural field conditions, an approach necessitated by low and spatially heterogeneous mite densities. Pooling likely reduced individual-level variation and may have masked ratio differences observed at the host level. Although this interpretation cannot be empirically validated within the constraints of the current field design, the convergence of viral ratios in mites despite pronounced plant-level differences suggests that vector-associated processes may buffer variation in virus proportions following acquisition.

Virus acquisition by arthropod vectors is not always proportional to host tissue titers and can be strongly virus-specific, reflecting differences in uptake efficiency, retention, or stability within the vector [33]. In wheat curl mites, differential transmission efficiencies among viruses and mite populations have been documented, indicating that vector-mediated processes can independently regulate virus abundance after acquisition [13]. In addition, asymmetry in replication and accumulation dynamics during mixed WSMV–TriMV infections has been demonstrated under controlled conditions and could extend to within-vector persistence [31].

Together, these observations suggest that convergence of viral proportions in mites may result from selective acquisition thresholds, differential viral retention, or constrained within-vector stability, rather than from simple averaging alone. Importantly, the present field-based design does not allow discrimination between processes occurring during virus acquisition and those operating after acquisition within the vector. Because mites were collected after feeding and analyzed as pooled samples, it remains unclear whether convergence of WSMV-to-TriMV ratios occurs at the point of ingestion from host tissues or arises from post-acquisition bottlenecks within the mite. Resolving this distinction will require future studies integrating individual-mite quantification with controlled time-course acquisition and retention experiments. From a management perspective, this pattern is significant, as it indicates that even when resistant cultivars reduce overall viral accumulation, surviving mites may still harbor balanced viral loads capable of initiating severe disease in subsequent susceptible crops or volunteer hosts that maintain the green bridge.

## 5. Conclusions

This study highlights the dynamic complexity of the WSM disease complex under field conditions. Interactions between WSMV and TriMV are strongly influenced by host genotype, vector biology, and environmental context. Cultivars carrying the *Wsm1* gene provided enhanced protection in the Texas Panhandle environment by restricting TriMV accumulation, thereby reducing overall disease severity even when WSMV remained detectable. The density-dependent relationship between plant and mite viral titers reinforces the central role of host resistance in reducing landscape-level inoculum pressure. Furthermore, the consistent WSMV-to-TriMV ratio observed in mites across cultivars suggests possible biological constraints on viral acquisition or within-vector regulation. Together, these findings advance understanding of virus–vector–host interactions in WSM and provide mechanistic insight to inform breeding strategies and integrated disease management programs.

## Data Availability

The raw data from this study will be made available upon request to the corresponding author.

## Supplementary Materials

The following supporting information can be downloaded at: https://www.mdpi.com/article/10.3390/insects17050459/s1, Table S1: qRT-PCR assay details and standard curve performance metrics for absolute quantification of WSMV and TriMV; Table S2. Mixed-effects model and post hoc contrast results for WSMV and TriMV accumulation during the 2021–2022 and 2022–2023 field seasons; Table S3: Supported post hoc outcomes from the same models.
