# Supplementary material for: Field Evidence for Asymmetric Regulation of Wheat Streak Mosaic Virus and Triticum Mosaic Virus Across the Wheat–Wheat Curl Mite Interface
Source: Insects. 2026 Apr 28;17(5):459. doi: 10.3390/insects17050459 (PMC13207605; doi:10.3390/insects17050459)
Supplement: Supplementary file 1 [file insects-17-00459-s001.zip › insects-4189434-supplementary.pdf]

insects

Article

Field Evidence for Asymmetric Regulation of Wheat Streak Mosaic Virus and Triticum Mosaic Virus Across the Wheat–Wheat Curl Mite Interface

Saurabh Gautam [1] and Kiran R. Gadhave [2,3,*]

[1] Alliance of Pest Control Districts, Tulare, CA 93274, USA; saurabh@apcd.ca.gov
[2] Texas A&M AgriLife High Plains Research and Extension Center, Canyon, TX 79015, USA
[3] Department of Entomology, Texas A&M University, College Station, TX 77843, USA; kiran.gadhave@ag.tamu.edu

* Correspondence: kiran.gadhave@ag.tamu.edu; Tel.: (806) 354-5806

Academic Editor: Ivan Milosavljević

Received: 19 February 2026
Revised: 15 April 2026
Accepted: 25 April 2026
Published: 28 April 2026

Copyright: © 2026 by the authors. Submitted for possible open access publication under the terms and conditions of the Creative Commons Attribution (CC BY) license.

Supplementary Table S1. qRT-PCR assay details and standard curve performance metrics for absolute quantification of WSMV and TriMV.

Virus	Target gene[1]	Primer name	Primer sequence (5′–3′)	Amplicon size (bp)	Amplification efficiency (%)	R^2	LOD (copies per reaction)	LOQ (copies per reaction)
WSMV	Coat protein (CP)	WSMV-F	AAGTGCAGAACAGCGTTG	138bp	90-110%	>. 99	10	100
		WSMV-R	AAACTGTGCGTGTTCTCC					
		WSMV-Probe	ACTGAGTGCGGGTACTAATGAGGAC					
TriMV	Coat protein (CP)	TriMV-F	CTTTGGAGCTTTAGCCTACC	138bp	90-110%	>.99	10	100
		TriMV-R	CTGGTCCACTGTCACAAA					
		TriMV-Probe	CAAGCCAATTATACCGCACAACGC					

[1] Primer sequences target conserved regions of the respective coat protein (CP) genes and were previously validated for specificity and efficiency. Standard curves were generated using ten-fold serial dilutions of plasmids containing the viral CP gene fragments (10^8–10^1 copies). Amplification efficiency was calculated from the slope of the standard curve using the equation $E = (10^{-1/slope} - 1) \times 100$, and linearity was assessed by the coefficient of determination (R^2).
The limit of detection (LOD) was defined as the lowest copy number consistently detected in ≥ 95% of technical replicates in a standard run, and the limit of quantification (LOQ) as the lowest copy number with a coefficient of variation ≤ 35% that were successfully detected in plant and/or mite samples.. All samples included in statistical analyses exceeded the LOQ.

Supplementary Table S2. Mixed-effects model and post hoc contrast results for WSMV and TriMV accumulation during the 2021–2022 and 2022–2023 field seasons

Season	Virus	Fixed effect	Num df	Den df	F value	p value
**2021–2022**	WSMV	Cultivar	2	48	4.32	0.018
		Sampling week	6	240	36.91	<0.001
		Cultivar × week	12	240	2.17	0.015
	TriMV	Cultivar	2	48	18.74	<0.001
		Sampling week	6	240	22.56	<0.001
		Cultivar × week	12	240	3.08	<0.001
**2022–2023**	TriMV	Cultivar	2	16	9.84	0.0017
		Sampling week	2	16	1.12	0.35
		Cultivar × week	4	16	0.88	0.49
	WSMV	Cultivar	2	16	2.41	0.12
		Sampling week	2	16	0.97	0.40
		Cultivar × week	4	16	0.71	0.59

Summary of mixed-effects model results evaluating effects of cultivar, sampling week, and spatial location on wheat streak mosaic virus (WSMV) and *Triticum mosaic virus* (TriMV) accumulation in wheat. Viral RNA copy numbers were log_{10}-transformed prior to analysis. For data analysis, block and flagged location nested within block were included as random effects. Location- and time-specific differences described below are supported by post hoc contrasts (estimated marginal means) derived from the same models.

Supplementary Table S3. Supported post hoc outcomes from the same models

Season	Virus	Scale of comparison	Significant pattern supported by post hoc contrasts
**2021–2022**	WSMV	Temporal (within cultivar)	WSMV accumulation changed significantly over time within cultivars, peaking mid-season and declining during senescence ($p < 0.05$)
		Spatial (among cultivars)	Cultivar differences were location- and time-specific; no consistent BT–Joe separation across all locations
	TriMV	Cultivar (field-wide)	BT (Wsm1) accumulated significantly lower TriMV than Joe and TAM 304 across most weeks and locations ($p < 0.05$)
		Temporal (within cultivar)	TriMV accumulation varied significantly across weeks within cultivars ($p < 0.05$)
**2022–2023**	WSMV	Cultivar × location × week	At a given flagged location and sampling week, WSMV titers differed significantly among cultivars, with TAM 304 > BT ≈ Joe ($p < 0.05$)
		Temporal (within cultivar, same location)	Within the same cultivar and flagged location, WSMV titers differed significantly among sampling weeks ($p < 0.05$)
	TriMV	Cultivar (field-wide)	BT accumulated significantly lower TriMV than Joe and TAM 304 across all three weeks ($p < 0.05$)
		Temporal (within cultivar)	TriMV accumulation differed significantly among sampling weeks within cultivars despite non-significant global time effect ($p < 0.05$)

https://doi.org/10.3390/insects17050459
